# Epidemiology on demand: population-based approaches to mental health service commissioning

**DOI:** 10.1192/pb.bp.114.047746

**Published:** 2015-10

**Authors:** James B. Kirkbride

**Affiliations:** 1 University College London

## Abstract

One in three people will experience a mental health problem in their lifetime, but the causes and consequences of psychiatric morbidity are socially patterned. Epidemiological studies can provide aetiological clues about the causes of disorder, and when they can provide robust estimates about risk in different strata of the population these can also be used translationally, to provide commissioners and service planners with detailed information about local service need. This approach is illustrated using a newly developed population-level prediction tool for first-episode psychosis, PsyMaptic. Such public mental health prediction tools could be used to improve allocation of finite resources, by integrating evidence-based healthcare, public health and epidemiology together.

One in three people will experience a psychiatric health disorder in their lifetime, according to recent estimates from a whole-population epidemiological study in Denmark.^1^ Such burden is not shared ubiquitously across populations, but is highly patterned, be it by largely unknown, rare or non-specific genetic variants and abnormalities, observable phenotypes (age, gender, ethnicity) or environmental exposures (including, but not limited to, socioeconomic position, education, substance use, prenatal insults, childhood adversity, traumatic life events and neighbourhood social disadvantage). For example, the incidence of psychotic disorders is several times higher in some sociodemographic groups, such as certain migrant and ethnic minority groups,^2^ most notably among Black Caribbean and African groups in England^3^ and Moroccan and Surinamese groups in The Netherlands,^4^ where excess rates are around 3–5 times greater than in the background population. Rates also vary in highly replicable ways by age and gender, with young men particularly at risk of psychotic disorder.^5^

While some of this patterning may be stochastic^6^ (genetic risk under non-assortative mating) or under biological control (risk by age or gender), the risk sets for, and consequences of, psychiatric disorders will also be shaped by socially patterned forces, which are disproportionately likely to affect poorer, more marginalised and vulnerable members of society, who are likely to shoulder the burden of our psychiatric morbidity. In terms of risk, this patterning may arise as a function of exposure to adverse environmental factors (independent or arising from gene-environment correlation),^6^ genetic risk due to assortative mating,^7^ epigenetic modifications^8^ or the role of cognitive impairment on risk of psychiatric disorder (which probably lies on the causal pathway between genes, environment and disorder).^9,10^ The consequences of psychiatric morbidity are also subject to strong social patterning, meaning some sections of society might be doubly disadvantaged.^11^ For example, with respect to psychotic disorders, people may experience social decline or drift,^12^ probably beginning premorbidly and usually sustained after the onset of first-episode psychosis (FEP), as a consequence of the onset of psychotic symptoms, especially negative symptoms,^13^ and cognitive impairment.^14^ This decline may continue or become exacerbated after onset of disorder as a result of additional issues, including side-effects from medication and stigma and discrimination experienced by people following psychosis onset. Social isolation,^15^ unemployment^16^ and drift into more disadvantaged communities^17^ are likely to be commonplace, in addition to the deleterious, and possibly synergistic,^18^ risk associated with these exposures.

## Early intervention for psychosis

While we have yet to elucidate clear, specific aetiologies through which genetic and environmental factors operate to cause psychosis, epidemiological studies can provide reliable, accurate estimates about the risk of disorder in different communities, based on both incidence (risk) and prevalence (risk and consequences) of psychotic disorder.^5,19^ Such data should be a valuable resource for mental healthcare service commissioners, who must make difficult choices about the efficient and effective allocation of finite resources for mental and physical health disorders throughout the population. The visionary commissioning of early intervention in psychosis (EIP) services,^20^ for example, was highly concomitant with a public mental-health-based approach for psychotic illness, based on available evidence. Thus, arising from evidence that a longer duration of untreated psychosis was associated with worsening functional, clinical and social outcomes,^21-23^ EIP services sought to intervene early in the initial presentation of psychotic symptoms.^24,25^ This approach partly targeted improving the consequences of illness onset and was a universal public health measure, broadly aimed at the group for whom a first episode of psychosis was most common – people under 35 years old.^5^ Some EIP services also provided early detection of psychosis provision at the stage which precedes psychosis, termed clinical high risk state,^26^ which focuses on preventing transition to a first episode of psychosis.^27,28^ This approach used both selective (young people with a family history of psychosis) and indicated (young people with early signs and symptoms of psychosis including a decline in functioning) prevention criteria to manage risk of disorder.

Accumulating evidence suggests that EIP services provide benefits across a plethora of individual, healthcare and societal outcomes.^29-31^ They are seen favourably by young people experiencing psychotic symptoms,^32^ given the holistic service model that targets a range of domains, including mental and physical health, identity and well-being, family involvement and vocational support. They reduce the risk of compulsory treatment and suicide in young people with psychosis,^33,34^ and fewer people with psychosis in EIP services are unemployed than in standard mental health services,^16^ although this figure remains stubbornly high when compared with their population-based peers.^35^ There is also a strong economic argument for EIP services. It is estimated that the NHS would save up to £44 million per year from fewer in-patient admissions if EIP were fully deployed,^36^ and there is consistent evidence that EIP provides long-run, sustained economic incentives over standard care;^37-40^ there is evidence that every pound invested in EIP services results in £18 of downstream savings.^36^ Psychotic disorders, more generally, also have pernicious effects on society. This can be measured acutely via lost economic productivity, with disorder onset typically coinciding with the age at which people have just completed their educational or vocational training and are about to enter the labour market.^41^ In the most severe cases, lost or reduced economic productivity may persist across the entire working age. The total societal cost of psychotic disorders in England has been estimated at £11.8 billion per year.^35^ By keeping more people in employment and improving other social outcomes,^29^ EIP services will provide long-term benefits to individuals, the economy and society over time.

Despite the strong rationale for such services, EIP have not been universally accepted or implemented.^42^ Services have faced a number of criticisms (see McGorry et al^43^ for an introduction), some better supported than others, including a lack of sufficient evidence for individual benefit when care is not sustained,^44^ cherry-picking of ‘easier’ cases,^45^ inadequate flexibility of EIP service delivery in rural communities,^46^ diversion of resources from standard mental healthcare services,^42^ case-loads being either below^47^ or in excess of government targets,^48^ and delays in treatment within mental health services in some regions threatening to jeopardise the very purpose of early intervention.^34,49,50^ This background of criticism has coincided with an increasingly difficult commissioning landscape^51^ where, despite ring-fenced NHS expenditure, real-term cuts to mental health services of 2% have been particularly keenly felt in EIP services, which have come to be viewed in some trusts as an unaffordable luxury.^34^ A recent audit by the mental health charity Rethink found that 50% of EIP services have experienced healthcare cuts in the past year alone, with a parallel perception by staff that the quality of service has also been reduced.^34^ Continued removal, reduction or restructuring of EIP services now threatens to undermine one of the National Health Service's (NHS's) exemplar models of integrated healthcare,^52^ at a time when other areas of healthcare delivery are moving towards such models.^53^ Indeed, this perverse logic runs counter to National Institute for Health and Care Excellence (NICE) recommendations that EIP should be provided for everyone in their first episode of psychosis, irrespective of age.^52^

## Towards integrated healthcare

Since the long-term clinical, social and economic benefits of EIP are most likely to be achieved when a full EIP package is implemented,^54^ which includes providing physical health checks and supported employment opportunities, clinical commissioning groups (CCGs) should favour full-fidelity EIP models.^52,55^ This is undoubtedly challenging in stringent economic times, when commissioners must make difficult decisions about the allocation of a finite set of resources across the spectrum of healthcare services. While piecemeal implementation of EIP services may provide the illusion of integrated healthcare for young people with severe mental health problems, piecemeal solutions are only likely to deliver piecemeal results, leaving services as precariously positioned to deliver expected results as they currently find themselves.

**Fig. 1:**
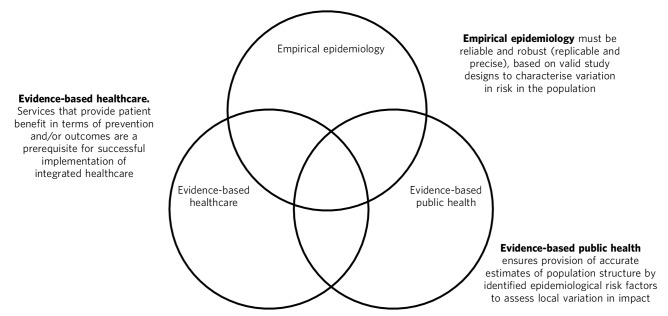
Three dimensions required for evidence-based integrated healthcare.

The integrated healthcare model envisaged by the Department of Health has the potential to offer an alternative approach to difficult decisions about resource allocation across health and social services,^53^ intractably founded on the fundamental principle of evidence-based decision-making. I suggest there are three aspects of the evidence base that need appraisal and integration to maximise the efficiency and effectiveness of resource allocation in an integrated healthcare model (Fig. 1). First, reliable and robust evidence about the epidemiological characteristics of any given disorder are required to understand whether and how risk varies between different members of the population, with a view to identifying those groups who are at greatest risk (empirical epidemiology). Second, services and treatments that have been shown to provide patient benefit in terms of prevention, reduced relapse or re-admission or improved quality of life and clinical and social prognosis must exist (evidence-based healthcare). Such care packages should also ideally have demonstrable cost-effectiveness over the medium- to long-term. Finally, a precise understanding of local population characteristics is required to understand how epidemiological risk translates into the public health impact of different disorders in different populations (evidence-based public health). An understanding of local needs is seen as central to the government's move towards integrated health,^53,56^ particularly as, with the exception of psychosis, less than a quarter of people experiencing mental health disorders are likely to be receiving any kind of treatment.^56-58^ This will only be achieved if the Department of Health, working alongside CCGs, can integrate these three strands of the evidence base to develop a detailed understanding of the multifaceted needs of local populations, and thereby allocate finite resources as efficiently as possible in response to dynamic local health issues.

## Evidence-based EIP services

With respect to EIP services, an integrated evidence base has allowed us to develop, validate and refine an online planning tool for the prediction of FEP at the population level, based on local need. The prediction tool combines empirical estimates about the risk of developing psychotic illness by major sociodemographic and environmental factors,^5,59^ such as age, gender, ethnicity and population density, with information about the population structure of different local authorities in England and Wales. This gives rise to the expected number of new FEP cases that would occur in a given population each year, providing commissioners with guidance on likely resource needs for psychotic disorders. We have made predictions freely available for every local authority in England and Wales, broken down by age and gender, in an online repository known as PsyMaptic (Psychiatric Mapping Translated into Innovations for Care, www.psymaptic.org). The predictions from the tool have been validated in East Anglia^60^ by comparing the predicted number of FEP cases with those empirically observed in a population at risk of nearly 1.4 million people over 2.5 years. We have recently published a revised version of this tool (version 1.1), which makes several important updates to improve its predictive accuracy (Table 1). Importantly, the new version simultaneously accounts for the effects of population density and socioeconomic deprivation,^61^ both of which are associated with the incidence of psychotic disorders, is validated over a longer period (3.5 years), and uses the latest population statistics for England and Wales, estimated from the 2011 census.

**Table 1:** Model comparisons between PsyMaptic versions 0.5 and 1.1

	Version 0.5	Version 1.1
Models tested	7	36

Denominator source	2009 mid-year census estimates	2011 census

Observation period, years	2.5	3.5

Person-years at risk (16–35 years)	1397 305	2 021 663

Minimum level of geography	Local authority	Local authority

Best-fitting model covariates	Age group, gender, age* sex interaction, ethnicity, population density	Age group, gender, age* sex interaction, ethnicity, population density, extent of deprivation, quadratic for extent of deprivation

Observed FEP cases (ICD-10), *n*	522	676

Predicted FEP cases (ICD-10), *n* (95% CI)	508 (459, 559)	667 (610, 722)

Equivalised RMSE (EIP level)^a^	19.0	16.3

Equivalised RMSE (LAD level)^a^	7.8	6.4

EIP correct (*n* = 6)^b^, *n*	5	5

LAD correct (*n* = 21)^b^, *n*	19	19

FEP, first-episode psychosis; EIP, early intervention psychiatry; LAD, local authority district; RMSE, root mean squared error.

a. RMSE gives a measure of how closely each predicted value was to the observed value, either at LAD or EIP level. Lower scores indicate better model fit. Versions 0.5 and 1.1 used different denominators and direct comparisons between the original RMSE values for version 0.5 (published in Kirkbride *et al*^60^) and version 1.1 were not possible, so equivalised RMSE values for model 0.5 are presented based on the denominator used in model 1.1.

b. The number of times the observed value fell within the 95% CIs of the prediction at EIP level (out of 6) or LAD level (out of 21). Both models perform equivocally at LAD and EIP levels in terms of number correctly predicted. However, the lower overall RMSE scores for model 1.1 provide clear evidence of improved fit, favouring model 1.1.

## Population-level psychosis prediction

Some of the aforementioned criticisms of EIP implementation (such as shortfalls or overestimates of expected case-loads) may have arisen as a direct result of the lack of tools to inform healthcare planners and commissioners about variation in need for services at the population level. Our tool overcomes part of this challenge by providing epidemiology ‘on demand’, centred on local population need and underpinned by a robust evidence base for FEP. It is important to recognise that PsyMaptic is only one of a suite of health informatics that commissioners will require to make effective decisions about the provision of local mental healthcare. For example, PsyMaptic predicts the expected incidence of ICD-10 clinically relevant FEP (F10–33), as confirmed by detailed OPCRIT review of case notes (http://sgdp.iop.kcl.ac.uk/opcrit/). It does not currently predict the additional resources required by EIP services to manage referrals who may present with underlying psychopathology, but require signposting to other, more appropriate services. Other data, such as the National Mental Health Minimum Dataset, which more accurately reveal all service use (not limited to those meeting clinical threshold for disorder), should be used in conjunction with such tools to inform commissioners about the probable additional burden of non-psychotic clinical psychopathology that EIP services may see, but were not originally provided for in the Policy Implementation Guide.^20^ It should be apparent that this problem becomes greater the earlier one tries to intervene, since early prodromal symptoms may be transitory or have relatively low specificity to later psychotic disorder.^62^ The recent trend in some CCGs to re-organise services around a clinical staging approach, with EIP services superseded by generalised youth mental health services,^63^ might be a service-side response to this phenomenon, but the non-specific (and perhaps non-clinical) nature of some early mental health symptoms will be a challenge for delivering effective, evidence-based youth mental healthcare, particularly where, for justifiable clinical and social reasons, services may delay formal diagnosis. We recommend that service commissioners use PsyMaptic as one part of a suite of evidence-based information available to them.

PsyMaptic provides proof-of-concept that empirical psychiatric epidemiology can be used to inform mental health service provision and public mental health. Predictions are prone to error, and we welcome observations from services where the tool performs well and where it does not, to enhance future versions. If similar forecasting could be applied to other mental or physical health disorders which have a robust empirical epidemiology, CCGs would have more complete information on which to make funding decisions across all health services in their locality, helping to drive the important demand for parity of esteem between physical and mental health.^64^ Fortunately, a growing range of tools is becoming available for services, CCGs and the Department of Health to make evidence-based decisions. PsyMaptic is one of a number of health indicators being used by Public Health England. For example, community mental health profiles,^65^ which detail the prevalence of various mental health disorders as well as risk factors and the wider determinants of health, are available for all local authorities in England. A further tool, by UCL Partners, is providing comprehensive mental health needs assessments,^66^ drawing on a range of data sources and providing estimates of local economic savings from intervention, including those for FEP and clinical high-risk states.

## Conclusions

Translational epidemiological tools have the potential to arm commissioners with evidence to allocate increasingly finite resources more efficiently across populations, centred on local need. The Health and Social Care Information Centre already publishes public mental health statistics which provide relatively comprehensive data for secondary mental healthcare. However, this information is not routinely combined with local estimates of variation in the incidence of different mental health disorders, using tools such as PsyMaptic (currently restricted to psychotic disorders). This synthesis would then allow for the potential size of the local unmet mental health need to be estimated, which can then be used to effectively inform local joint strategic needs assessments (JSNAs). This in turn informs commissioning and health and well-being board strategies. Therefore, routine inclusion of such information in JSNAs could have a very large role in reducing the size of mental health unmet need.
